# Thin Sectioning of Slice Preparations for Immunohistochemistry

**DOI:** 10.3791/194

**Published:** 2007-04-28

**Authors:** Jae-Joon Park, Miles G. Cunningham

**Affiliations:** Department of Medicine, Yonsei University College of Medicine, Severance Hospital; Department of Psychiatry, Harvard Medical School

## Abstract

Many investigations in neuroscience, as well as other disciplines, involve studying small, yet macroscopic pieces or sections of tissue that have been preserved, freshly removed, or excised but kept viable, as in slice preparations of brain tissue.  Subsequent microscopic studies of this material can be challenging, as the tissue samples may be difficult to handle.  Demonstrated here is a method for obtaining thin cryostat sections of tissue with a thickness that may range from 0.2-5.0 mm.  We routinely cut 400 micron thick Vibratome brain slices serially into 5-10 micron coronal cryostat sections.  The slices are typically first used for electrophysiology experiments and then require microscopic analysis of the cytoarchitecture of the region from which the recordings were observed.  We have constructed a simple device that allows controlled and reproducible preparation and positioning of the tissue slice.  This device consists of a cylinder 5 cm in length with a diameter of 1.2 cm, which serves as a freezing stage for the slice.  A ring snugly slides over the cylinder providing walls around the slice allowing the tissue to be immersed in freezing compound (e.g., OCT).  This is then quickly frozen with crushed dry ice and the resulting  wafer  can be position easily for cryostat sectioning.  Thin sections can be thaw-mounted onto coated slides to allow further studies to be performed, such as various staining methods, in situ hybridization, or immunohistochemistry, as demonstrated here.

**Figure:**
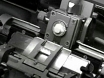


## Protocol

Prepare mold from tape for OCT platform.
Fill mold with OCT. Freeze within cryostat or by using crushed dry ice.
Remove tape from around frozen OCT platform.
Align marks on freezing chuck and cryostat mounting stage and lock in chuck.
Section through OCT platform until surface is flat.
Remove resurfaced OCT platform and place on cryostat freezing stage.
Place tissue sample (previously cryopreserved with 30% glycerol or sucrose in PBS) in OCT.
Prepare freezing column with outer ring projecting about 5 mm above top of column forming well for OCT.
Carefully position tissue sample onto center of freezing column surface and slowly add OCT until well is filled.
Surround freezing column with crushed dry ice. Tissue and OCT should completely freeze within 20-60 seconds.
As preparation increases in temperature, the outer ring can be removed while the sample remains frozen.
Slide sample off freezing column sideways and place in cryostat.
Place drop of OCT on surface of OCT platform and position specimen (tissue down) applying firm pressure. Specimen will quickly freeze onto OCT platform.
Secure chuck onto cryostat mounting stage with marks aligned.
Section through OCT superficial to the tissue specimen.
Thaw mount thin sections onto glass slides and store frozen or at room temperature.
Immunoreactions can be performed for tissue mounted on glass slides.
Reagent is pooled onto slide, can be gently agitated, and may be covered if light-sensitive.
Subsequent stages of the reaction are easily performed by inverting slide into waste receptacle, wicking the slide, and then applying the next reagent.

## Discussion

The protocol presented here provides researchers with a concise, easy-to-follow outline of how to obtain thin cryostat sections of small, difficult-to-manage, tissue pieces, such as biopsies and brain slices for further studies to be performed, such as various staining methods, in situ hybridization, or immunohistochemistry.
